# The role of vitamin D in pulmonary disease: COPD, asthma, infection, and cancer

**DOI:** 10.1186/1465-9921-12-31

**Published:** 2011-03-18

**Authors:** Christian Herr, Timm Greulich, Rembert A Koczulla, Silke Meyer, Tetyana Zakharkina, Meret Branscheidt, Rebecca Eschmann, Robert Bals

**Affiliations:** 1 Department of Internal Medicine, Division for Pulmonary Diseases, Philipps-Universtät Marburg, 35043 Marburg, Germany; 2 Department of Internal Medicine, Division of Endocrinology & Diabetology, Department of Internal Medicine, University Hospital Marburg, 35043 Marburg, Germany; 3 Department of Pulmonology, University of the Saarland, 66421 Homburg Saar, Germany

**Keywords:** Vitamin D, mortality, asthma, COPD, respiratory tract infection, immunity

## Abstract

The role of vitamin D (VitD) in calcium and bone homeostasis is well described. In the last years, it has been recognized that in addition to this classical function, VitD modulates a variety of processes and regulatory systems including host defense, inflammation, immunity, and repair. VitD deficiency appears to be frequent in industrialized countries. Especially patients with lung diseases have often low VitD serum levels. Epidemiological data indicate that low levels of serum VitD is associated with impaired pulmonary function, increased incidence of inflammatory, infectious or neoplastic diseases. Several lung diseases, all inflammatory in nature, may be related to activities of VitD including asthma, COPD and cancer. The exact mechanisms underlying these data are unknown, however, VitD appears to impact on the function of inflammatory and structural cells, including dendritic cells, lymphocytes, monocytes, and epithelial cells. This review summarizes the knowledge on the classical and newly discovered functions of VitD, the molecular and cellular mechanism of action and the available data on the relationship between lung disease and VitD status.

## 

VitD supplementation appears to be correlated with decreased total mortality [1]. In the early 1920s a group of scientists independently discovered that irradiating of certain foods with ultraviolet light renders them antirachitic [2,3] and in 1922 Elmer V. McCollum identified an antirachitic substance in cod liver oil and called it "vitamin D" [4]. While the role of VitD in calcium and bone homeostasis has been well described, its activities on other physiological and pathophysiological processes have been recognized only in the last years. Epidemiological data suggest that several lung diseases, all inflammatory in nature, may be related to activities of VitD. VitD deficiency might have a role in the development of these diseases. The underlying mechanisms how VitD metabolisms could be linked to the pathophysiology of these diseases are often complex and not fully understood. This review summarizes the role of VitD in lung diseases.

## Evolutionary aspects

VitD and its receptors are found throughout the animal kingdom and are often linked to bone and calcium metabolisms. The fact that precursors of VitD are found in ancient organisms like krill and phytoplankton that existed unchanged for at least 750 million years [5] highlights its importance in physiologic and homeostatic processes.

Variants of VitD and its receptors have been identified in higher terrestrial vertebrates like humans [6], rodents [7], birds [8], amphibia [9], reptiles [10], as well as in zebrafish [11]. These animals possess a calcified skeleton and depend on a functional VitD hormone system for calcium and phosphorus homeostasis. Surprisingly, functional VitD receptors (VDRs) have also been found in lampreys, an ancient vertebrate that lacks a calcified skeleton [12]. VDRs were also identified in animals with a naturally impoverished VitD status like the subterranean mole rat [13] and a frugivorous nocturnal mammal, the Egyptian fruit bat Cavaleros [14]. VitD precursors have been found in ancient organisms like phytoplankton and zooplankton, some of which exist unchanged for at least 750 million years [5,15]. Functional VitD hydroxylases have also been characterized in bacteria like strains of *actinomyces *[16,17] and *streptomyces *[18,19]. The precursors of VitD in those organisms may function as a natural sunscreen to protect the host against UV-radiation, since the absorption spectra of pro-vitamin D and their photoproducts overlap with the absorption maxima of DNA, RNA, and proteins [20].

## Role of VitD in bone metabolism

**Figure 1:**
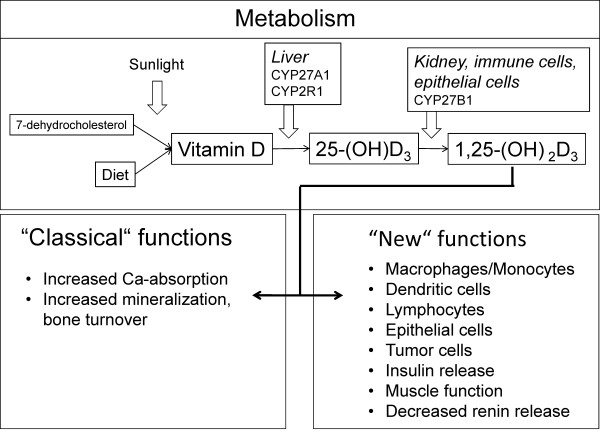
**Metabolism and effects of VitD**. VitD can be obtained from food or from synthesis in the skin under exposure to light. The precursor is hydroxylated cytochrome P450 25-hydroxylase enzymes CYP27A1 and/or CYP2R1 and subsequently by the cytochrome P450 enzyme 25-hydroxyvitamin D-1α-hydroxylase (CYP27B1) and converted to the bioactive 1,25-(OH)_2_D_3_, which has role in Ca and bone metabolism and, in addition, in several other biological processes. Of note, bioactive 1,25-(OH)_2_D_3 _can also be generated in lung epithelia cells and monocytes/macrophages.

VitD, which is photosynthesized in the skin or has been derived from nutrition, is metabolized two times, before it mediates its calcemic effects by binding to the nuclear VitD receptor (VDR) [21,22](Figure 1). The metabolizing enzymes belong to a group of cytochrome P450 hydroxylases, which can be found in eukaryotes, bacteria, fungi and plants. In the human liver, the first hydroxylation of VitD on C-25 is performed by mitochondrial 25-hydroxylase enzymes (gene names: CYP27A1 [23] and/or CYP2R1 [24]) that both belong to the cytochrome P450 family. The inactive 25-(OH)-vitamin D_3 _(25-(OH)D_3_) metabolite is further hydroxylated at position 1α by the mitochondrial cytochrome P450 enzyme 25-hydroxyvitamin-D-1α-hydroxylase (gene name: CYP27B1) and converted to the bioactive 1α,25-dihydroxyvitamin D(1,25-(OH)_2_D_3_). This latter step is mainly localized to the proximal kidney tubule [25], however, many other cell types, including lung epithelial cells, are capable to perform this reaction [26-29]. The serum concentration of 25-(OH)D_3 _reflects the organism's VitD supply [30]. In the blood, VitD and the inactive, relatively stable 25-(OH)D_3 _metabolite are bound in 99% to the vitamin D binding protein (DBP) [31]. DBP polymorphisms (Gc phenotype) are related to the DBP concentration and VitD status [32]. The 1α-hydroxylation of 25-(OH)D_3 _is upregulated by parathyroid hormone (PTH), calcitonin, low calcium- and phosphate levels as well as by estrogen, prolactin and growth hormone [33]. Calcitonin, cortisol, high phosphate levels and 25-(OH)D_3 _suppress the 25-hydroxyvitamin D-1α-hydroxylase activity [34]. 1,25-(OH)_2_D_3 _itself works as its own negative feedback regulator by induction of the expression of a 24-hydydroxylase (CYP24A1). Further, 1,25-(OH)_2_D_3 _decreases the production and secretion of PTH. PTH synthesis and secretion is induced by decreased serum calcium levels, which are detected by the calcium sensing receptor of the parathyroid gland. PTH effects renal tubular reabsorption of calcium, renal production of 1,25-(OH)_2_D_3 _and promotes osteoclastogenesis [35].

1,25-(OH)_2_D_3 _is essential for the development and maintenance of the growth plate, chondrocyte growth, and the mineralised bone [21]. 1,25-(OH)_2_D_3 _modulates the osteoclastogenesis by regulation of the receptor activator of nuclear factor kappa B (RANK), RANK ligand (RANKL) and the soluble receptor osteoprotegerin (OPG) [36]. It increases the expression of RANKL on the osteoblast surface, which supports maturation of progenitor and mature osteoclasts, and it inhibits OPG expression, which binds RANKL and prevents RANK mediated osteoclastogenesis [37].

VitD deficiency causes the development of an imbalanced calcium- and phosphate-homeostasis and the occurrence of the bone diseases osteopenia, osteoporosis, rickets, and osteomalacia with a subsequently increased fracture risk [38]. The 25-(OH)D_3 _serum concentration is directly associated with bone mineral densitys. VitD deficiency has several causes including inadequate sun exposure (and loss of functional capacity of the skin especially in the elderly), limited renal and hepatic function or insufficient intestinal resorption [39]. In VitD deficiency, the feedback on the PTH gene promoter is lacking resulting in parathyroid hyperplasia, hyperparathyroidism, and a mineralization defect of the bone.

1,25-(OH)_2_D_3 _regulates many target genes by binding to the VDR: approximately 3% of the mouse and human genome is regulated via the VitD pathway [40]. As non-genomic action of VitD in chondrocytes, it increases the membrane-lipid turnover, prostaglandin production and protease activity, leading to bone matrix modification and calcification. Additionally to the expression of VDR in bone and multiple tissues, the presence of 1α-hydroxylase in cells of several extrarenal tissues such as bone as well as skin, prostate, the respiratory and gastrointestinal tract, strongly suggest that VitD impacts on processes beyond the calcium and bone metabolism.

## Role of VitD in immunity and host defense

More than a century ago (1849), the British physician C.J.B. Williams described the use of cod liver oil in the treatment of tuberculosis. He reported that among his tuberculosis patients, 206 out of 234 showed a "marked and unequivocal improvement" after treatment with cod liver oil [41]. Since then manifold functions of VitD have been discovered, indicating that VitD regulates many cellular processes and is potentially involved in the development of many diseases. Since the discovery of VDRs in a variety of cells of the adaptive immune system such as B- and T-lymphocytes [42,43], there have been numerous reports about the immunomodulatory activities of VitD.

Cellular studies revealed that VitD modulates the activity of various defense and immune cells including monocytes, macrophages, lymphocytes, or epithelial cells:

• Monocytes/macrophages: Low serum concentrations of VitD in patients with rickets correlate with decreased phagocytic activity of macrophages [44] that could be reversed by supplementation with 1,25-(OH)_2_D_3 _[45]. Antimicrobial activity of macrophages against *M. tuberculosis *is increased in the presence of 25-(OH)D_3 _after stimulation with mycobacterial ligands. Mycobacterial activation of toll-like receptor-2 (TLR-2) leads to an increased expression of VDR and CYP27B that results in an increased conversion of 25-(OH)D_3 _to 1,25-(OH)_2_D_3 _and subsequent expression of the antimicrobial peptide cathelicidin via VDR [46,47].

• B lymphocytes: It has been shown that 1,25-(OH)_2_D_3 _plays a role in B cell homeostasis by the inhibition of proliferation and induction of apoptosis of activated B cells [48]. 1,25-(OH)_2_D_3 _inhibits the differentiation of B lymphocytes to plasma cells and memory B cells. These mechanisms may contribute to the pathogenesis of B-lymphocyte related diseases like systemic lupus erythematosus (SLE). Patients with SLE have significant lower serum concentration of both 25-(OH)D_3 _and 1,25-(OH)_2_D_3 _[49,50].

• T lymphocytes: A well-established function of VitD within the adaptive immune system is its ability to modulate T lymphocyte proliferation and function. The biologically active 1,25-(OH)_2_D_3 _inhibits proliferation of T_H _lymphocytes [51] and shifts the expression of cytokines from a T_H_1 based response towards a T_H_2 based profile [52,53]. Although 1,25-(OH)_2_D_3 _might be able to involve direct effects on T lymphocytes through the support of differentiation of regulatory T cells, current data indicate that 1,25-(OH)_2_D_3 _exerts its influence on the adaptive immune response by modulating the functions of dendritic cells (DCs). Regulatory T cells seem to be activated by VitD with skewing of the Th1/Th2 balance towards Th2 [54]. Of note, there is evidence for and against the role of VitD in Th2 biased diseases [55], which will be discussed in more detail in the asthma section below.

• Dendritic cells: The response of DCs to 1,25-(OH)_2_D_3 _is restricted to myeloic DC, that express a different set of TLRs and cytokines than plasmacytoic DCs, which showed no tolerogenic response to 1,25-(OH)_2_D_3 _[56]. 1,25-(OH)_2_D_3 _inhibits the maturation of DCs and enhances the expression of cytokines like IL-10, thereby 1,25-(OH)_2_D_3 _induces tolerance through the suppression of T_H_1 lymphocyte development and the induction of regulatory T cells [57].

• Epithelial cells: Airway epithelial cell express enzymes of the VitD metabolism and are capable to convert the precursor 25-(OH)D_3 _into the active 1,25-(OH)_2_D_3 _from [29,58]. They are an important source of 1,25-(OH)_2_D_3 _that induces the expression of cathelicidin or CD14 by cells of the innate immune system. 1,25-(OH)_2_D_3 _converted by airway epithelial cells is able to modulate the inflammatory profile after a viral infection by blocking the poly(I:C) induced chemokine and cytokine production while maintaining the antiviral activity [28,59]. As epithelial cells are primary targets of respiratory pathogens and cathelicidin has antibacterial and antiviral activity, a seasonal decrease of VitD-dependent epithelial host defense could contribute to increased numbers of lower respiratory tract infection (RTI) during winter.

## Roles of VitD in pulmonary diseases

VitD has complex effects on pulmonary cell biology and immunity with impact on inflammation, host defense, wound healing, repair, and other processes. While the knowledge on direct mechanistic links between VitD and lung diseases is limited, a number of epidemiological and experimental are available that highlight the relevance of this connection.

### a) Asthma

A connection between VitD status and asthma has been considered since many years. VitD deficiency has been blamed as one cause of increased asthma prevalence in the last decades [60]. VDR variants were found to be associated with asthma in patient cohorts [61]. A recent clinical investigation showed that high VitD levels are associated with better lung function, less airway hyperresponsiveness and improved glucocorticoid response [62]. A population-based study suggested that lower VitD levels are associated with increased requirements for inhaled corticosteroids in children [63]. Vitamin D insufficiency is common in this children with mild-to-moderate persistent asthma and is associated with higher odds of severe exacerbation [64]. Epidemiologic studies have also shown that maternal VitD intake during pregnancy protects from wheezing in childhood [65,66]. In contrast, also data exist that children whose mothers had high VitD levels in pregnancy had an increased risk of eczema and asthma [67], suggesting that the time point of Vit D supplementation seems to determine the susceptibility to atopic disease. On the experimental level in a murine asthma model, the VDR is necessary for the development of an allergic airway inflammation [68].

The underlying mechanisms how VitD modulates the pathogenesis of asthma are not clear. VitD may protect from developing respiratory infections that could serve as trigger for a deterioration of asthma [69]. VitD may also modulate the function of various immune cells as outlined above. Interestingly, application of VitD is potentially capable to overcome the poor glucocorticoid responsiveness in severe asthmatics by upregulation of IL-10 production from CD4+ T cells [70].

### b) Chronic obstructive lung disease (COPD)

The connection between VitD status and COPD has attracted attention in the recent months. This is based on data from observational studies that determined levels of VitD in COPD patients. Black and colleagues examined data from the NHANES III data set (cross-sectional survey of 14091 adults in the US). After adjustment for potential confounders, a strong relationship between serum levels of VitD and lung function (FEV_1 _and FVC) was found [71]. Although a significant correlation with airway obstruction could not be found, the observed dose-response relationship may suggest a causal link [72]. A number of studies have reported on 25-(OH)D_3 _levels in COPD patients. Forli et al. found VitD deficiency (in this study defined as below 20 ng/ml) in more than 50% of a cohort waiting for lung transplantation [73]. In an outpatient study on patients with COPD in Denmark, 68% of the participants had osteoporosis or osteopenia [74]. A recent study showed that VitD deficiency is highly prevalent in COPD and correlates with variants in the VitD binding gene [75]. There are several factors that could account for VitD deficiency in COPD patients: Poor diet, a reduced capacity of aging skin for VitD synthesis, reduced outdoor activity and therefore sun exposure, an increased catabolism by glucocorticoids, impaired activation because of renal dysfunction, and a lower storage capacity in muscles or fat due to wasting [76]. Many steps of the VitD pathway (intake, synthesis, storage, metabolism) can potentially be disturbed in COPD patients.

A single nucleotide polymorphism (SNP) of the DBP was shown to be associated with a decreased risk of COPD by a mechanism that is unclear [77]. Similar SNPs in the gene coding for DBP may influence the level of circulating 25-(OH)D_3 _and 1,25-(OH)_2_D_3 _[32,78]. Therefore it has been hypothesized that their protective role might be mediated by the bioavailability of 1,25-(OH)_2_D_3 _[79].

The mechanisms that link VitD biology with the development of COPD are largely speculative:

1) The association of VitD deficiency and reduced lung function could depend on the calcemic effects of VitD. The vital capacity and total lung capacity was found to decline with an increasing number of thoracic vertebral fractures as a direct consequence of VitD deficiency [80]. Nuti et al. observed 3030 ambulatory COPD patients and found a strong association between COPD severity and fractures [81]. Kyphosis related to osteoporosis caused limitation in rib mobility and inspiratory muscle function and correlated with a reduction in FEV_1 _and FVC [82]. The altered properties of the thoracic skeleton could result in failure of the respiratory muscles contributing to the pathophysiology of COPD.

2) VitD deficiency could result in altered host defense of the lung with subsequent growth of an abnormal flora that triggers inflammation. Acute exacerbations of COPD are an important cause of hospitalization and lead to a faster decline in FEV_1 _[83]. Exacerbations are triggered by viruses, bacteria, atypical strains, or a combination of these [84-87]. Potential bacterial pathogens are detected in about 50% of exacerbations. A therapeutic consequence would be the up-regulation of the innate immune defense system. Wang and colleagues demonstrated that genes coding for the antimicrobial peptide cathelicidin (LL-37/hCAP-18) are regulated by VDRE-containing promoters [88]. In cultured monocytes, a local increase of the 1,25D3-VDR complex stimulates the production of LL-37, resulting in an improved intracellular eradication of *Mycobacterium tuberculosis *[47]. The data demonstrated that the activation of TLRs on human monocytes triggers a microbicidal pathway that is dependent on both the endogenous production and action of 1,25-(OH)_2_D_3 _through the VDR.

3) The effect of VitD on extracellular matrix homeostasis not only in bone tissue, but also within the lung may have a role in COPD development. Boyan et al. found VitD to be an autocrine regulator of extracellular matrix turnover and growth factor release via matrix metalloproteinases [89]. Matrix metalloproteinasis-9 (MMP-9) has been shown to be elevated in induced sputum of COPD patients and a causative role has been suggested in the development of COPD [90]. VitD also to attenuates TNF-alpha induced upregulation of MMP-9 in keratinocytes [91]. VitD deficiency may lead to a reduced attenuation of MMP-9 activity resulting in enhanced degradation of lung parenchyma.

Recently, it has been recognized that COPD is a systemic disease [92] with several closely related comorbidities [93]. Interestingly, VitD deficiency is associated with a equivalent spectrum of diseases including coronary heart disease, cancer, inflammatory disease and infection [76]. Comorbidities of COPD such as reduced bone mineral density and skeletal muscle weakness [94,95] have been associated with low VitD serum concentrations.

### c) Infection

#### Tuberculosis

A number of candidate polymorphisms of VitD receptor (VDR) and VitD binding protein (DBP) have been identified that modulate the development of tuberculosis [96]. The genotype tt (detected by Taq I digestion) is associated with decreased risk of tuberculosis. As described by Lewis et al. [97], larger studies are required to determine whether VDR polymorphisms play a role in genetic susceptibility to tuberculosis worldwide. In a recent meta-analysis, low serum levels of 25-(OH)D_3 _were associated with a higher risk of active tuberculosis. The pooled effect size was 0.68 with 95% CI 0.43 - 0.93. The authors concluded that the low VitD levels increase the risk of active tuberculosis [98]. There are several randomized, double-blind, placebo-controlled trials of VitD treatment in tuberculosis. In one study, 67 tuberculosis patients were randomized to receive VitD (0.25 mg/day) or placebo during the 6 initial week of Tb treatment [99]. A statistical significant difference in sputum conversion (i.e, the change of detectable to no detectable *Mycobacteria *in the sputum) was discovered in favor of the VitD group (100% vs. 76,7%; p = 0.002). Another trial was conducted in 192 healthy adult tuberculosis contacts in London, United Kingdom [100]. Participants were randomized to receive a single oral dose of 2.5 mg VitD or placebo and followed up at 6 weeks. VitD supplementation significantly enhanced the ability of participants' whole blood to restrict BCG-lux luminescence after 24 hours in vitro as compared with placebo, but did not affect antigen-stimulated IFN-gamma secretion after 96 hours. As the innate immune responses are mobilized more rapidly than acquired immune responses, the authors interpreted the 24- and 96-hour results as indicators of innate and acquired responses, respectively. They concluded that vitamin D supplementation may primarily enhance innate responses to mycobacterial infection. Wejse et al. included 365 tuberculosis patients starting anti-tuberculotic treatment in Guinea Bissau [101]. 281 patients completed the 12 month follow-up. The intervention was 100,000 IU cholecalciferol or placebo at inclusion and again at 5 and 8 months after start of treatment. Reduction in TBscore and sputum smear conversion rates did not differ among VitD and placebo treated patients. Taken those data together there seems to be a benefit of VitD in the treatment of tuberculosis but this could not be reproduced in the largest study so far.

#### Respiratory tract infections (RTI)

RTI are more common in the winter period than during summertime. Because the food intake of VitD is insufficient, sunlight exposure is the primary determinant of VitD status in humans, and seasonal differences in VitD level in human are well documented [76]. During the winter months, there is insufficient UV-B exposure to produce sufficient amounts of VitD. Wintertime VitD insufficiency may explain seasonal variation in influenza and other, mostly viral, RTIs [102]. Ginde et al. performed a secondary analysis of the Third National Health and Nutrition Examination Survey, hypothesizing an association between 25-(OH)D_3 _level and self-reported upper respiratory tract infections (URTI) in 18883 subjects [103]. After adjusting for season, body mass index, smoking history, asthma, and COPD, lower 25-(OH)D_3 _levels were independently associated with recent URTI. In patients with respiratory tract diseases (asthma and COPD) the association between 25-(OH)D_3 _level and URTI seemed to be even stronger (OR, 5.67 and 2.26, respectively). Avenell and colleagues used data from the RECORD trial (VitD in secondary prevention of osteoporotic fractures; *n *= 5292) [104]. In a "per protocol" analysis, a trend towards a benefit of VitD vs. placebo was detected, though not statistically significant. Despite the large number of patients in these studies, restrictions arise from the retrospective data analysis. A prospective cohort study included 800 young Finnish men serving on a military base [105]. Their serum 25-(OH)D_3 _was measured in the beginning of a 6 month observational period. Subjects with low 25-(OH)D_3 _levels had significantly more days of absence from duty due to respiratory infection than did control subjects (p = 0.004). In a case control study a total of 150 children (80 cases, 70 controls) was enrolled [106]. Low serum 25-(OH)D_3 _(≤ 22.5 nmol/l) was associated with a significantly higher odds ratio for having severe acute lower respiratory tract infections (p < 0.001). These studies support an role of VitD in the development of lung infection.

However, in a recent clinical trial, Li-Ng et al. randomized 162 adults to 50 μg VitD (2000 IU) daily or placebo for 12 weeks. Using a questionnaire they recorded the incidence and severity of upper RTI symptoms. Although VitD serum levels increased significantly in the VitD treated group (vs. no change in the placebo group), there was no benefit of VitD supplementation in decreasing the incidence or severity of symptomatic URTI [107]. This may be explained by the relatively low number of subjects. Furthermore, the time period of 12 weeks was probably too short to show any effect. Taken together, there is growing evidence for a protective role of VitD in the development of RTI but high quality randomized clinical trials within a sufficiently high number of patients and for a sufficient period of time are missing. In a recently published trial, the supplementation of 1500 E VitD per day resulted in deceases incidence of influenza A by 64% [69].

### d) Cancer

A number of studies suggest that low levels of VitD are associated with an up to 50% increased risk of colon, prostate, or breast cancer [76,108]. As an example, a recent nested case-control study showed that pre-diagnostic levels of VitD are inversely correlated with the risk of colon cancer [109]. For lung cancer, the picture is not clear at the present time. While TaqI polymorphism of the VDR gene appears to be a risk factor for lung cancer [110], low levels of VitD were only a cancer risk factor in subgroups, i.e., in women and young individuals [111]. In patients with diagnosed lung cancer, there was no main effect of VitD level on overall survival [112]. In preclinical animal models using carcinogen (NNK)-induced lung carcinogenesis, application of 1,25-(OH)_2_D_3 _resulted in decreased cancer growth [113].

## Conclusions

VitD has a number of activities in addition to its effect on calcium and bone homeostasis and influences process such as immune regulation, host defense, inflammation, or cell proliferation. VitD deficiency is potentially involved in a number of lung disease. Several hurdles must be overcome to validate the benefit of VitD-based therapies: 1) Basic mechanisms are not clear and the involved molecular pathways are likely difficult to identify because VitD impacts on a variety of biological processes in parallel. 2) Conclusive data from interventional studies are missing for many disease entities. 3) Since VitD has been used for many years, the pharmaceutical industry might hesitate in starting a development program. Nevertheless, the data available indicate that VitD could be beneficial for the prevention or therapy of important lung diseases.

## List of abbreviations

1,25-(OH)_2_D_3_: 1α: 25-dihydroxyvitamin D; 25-(OH)D_3_: D_3_25-(OH)-vitamin D_3_; TLR: toll like receptor; VitD: vitamin D;

## Competing interests

The authors declare that they have no competing interests.

## Authors' contributions

RB developed the concept of the review, all authors contributed in writing and reviewing the paper. All authors read and approved the final manuscript.
